# Electron Tunneling Rates in Respiratory Complex I Are Tuned for Efficient Energy Conversion**

**DOI:** 10.1002/anie.201410967

**Published:** 2015-01-19

**Authors:** Simon de Vries, Katerina Dörner, Marc J F Strampraad, Thorsten Friedrich

**Affiliations:** Department of Biotechnology, Institution Delft University of TechnologyJulianalaan 67, 2628 BC, Delft (The Netherlands); Institut für Biochemie, Albert-Ludwigs-Universität FreiburgAlbertstrasse 21, 79104 Freiburg (Germany)

**Keywords:** bioenergetics, electron tunneling, enzyme kinetics, metalloenzymes, reaction mechanisms

## Abstract

Respiratory complex I converts the free energy of ubiquinone reduction by NADH into a proton motive force, a redox reaction catalyzed by flavin mononucleotide(FMN) and a chain of seven iron–sulfur centers. Electron transfer rates between the centers were determined by ultrafast freeze-quenching and analysis by EPR and UV/Vis spectroscopy. The complex rapidly oxidizes three NADH molecules. The electron-tunneling rate between the most distant centers in the middle of the chain depends on the redox state of center N2 at the end of the chain, and is sixfold slower when N2 is reduced. The conformational changes that accompany reduction of N2 decrease the electronic coupling of the longest electron-tunneling step. The chain of iron–sulfur centers is not just a simple electron-conducting wire; it regulates the electron-tunneling rate synchronizing it with conformation-mediated proton pumping, enabling efficient energy conversion. Synchronization of rates is a principle means of enhancing the specificity of enzymatic reactions.

NADH:ubiquinone oxidoreductase, respiratory complex I, is the main entry point for NADH in mitochondrial and bacterial respiratory chains. The free energy of the redox reaction drives the translocation of four protons per NADH[1] (Figure 1), generating a proton motive force essential for energy-consuming processes. Complex I consists of a peripheral arm located in the aqueous milieu and a membrane arm embedded within the lipid bilayer. The peripheral arm catalyzes electron transfer from NADH to ubiquinone (Q) by a flavin mononucleotide (FMN) and a chain of seven iron–sulfur (FeS) centers (Figure 1). Another center, N1a, is located on the opposite side of the electron-transfer chain. The correlation between the structurally defined FeS centers and their EPR signals[2] was established by double electron–electron resonance experiments (Figure 1 B).[2b] The Q binding site is located at the interface of the two arms.[3] Based on the structural and functional data the coupling between electron transfer and proton translocation was proposed to be brought about by conformational changes upon reduction of N2 and Q and then transmitted to four proton channels (Figure 1).[3b,^4^] Electron transfer rates from NADH to Q, which includes the longest electron-tunneling distance of 14.1 Å between centers 4Fe[75]H and N4 (Figure 1), are 150–200 s^−1^ for the *E. coli* complex I.[5] Electron tunneling half-lives (*t*_1/2_) for this elementary step were estimated at 70 μs,[6] 95 μs,[7] and 275 μs,[8] respectively, depending on the boundary conditions of Marcus theory. All other half-lives were calculated as *t*_1/2_=25–400 ns, and *t*_1/2_=5–10 μs between centers N1b and 4Fe[75]C.[6–8] Recently, electron transfer in complex I was monitored by EPR spectroscopy of ultrafast freeze-quenched samples indicating a rapid reduction of N2 and N1a with *t*_1/2_≈60 μs[9] for the first NADH, followed by a slower reduction by the second NADH with *t*_1/2_≈1 ms for N1b and N4 due to slow dissociation of NAD^+^.[9]

**Figure 1:**
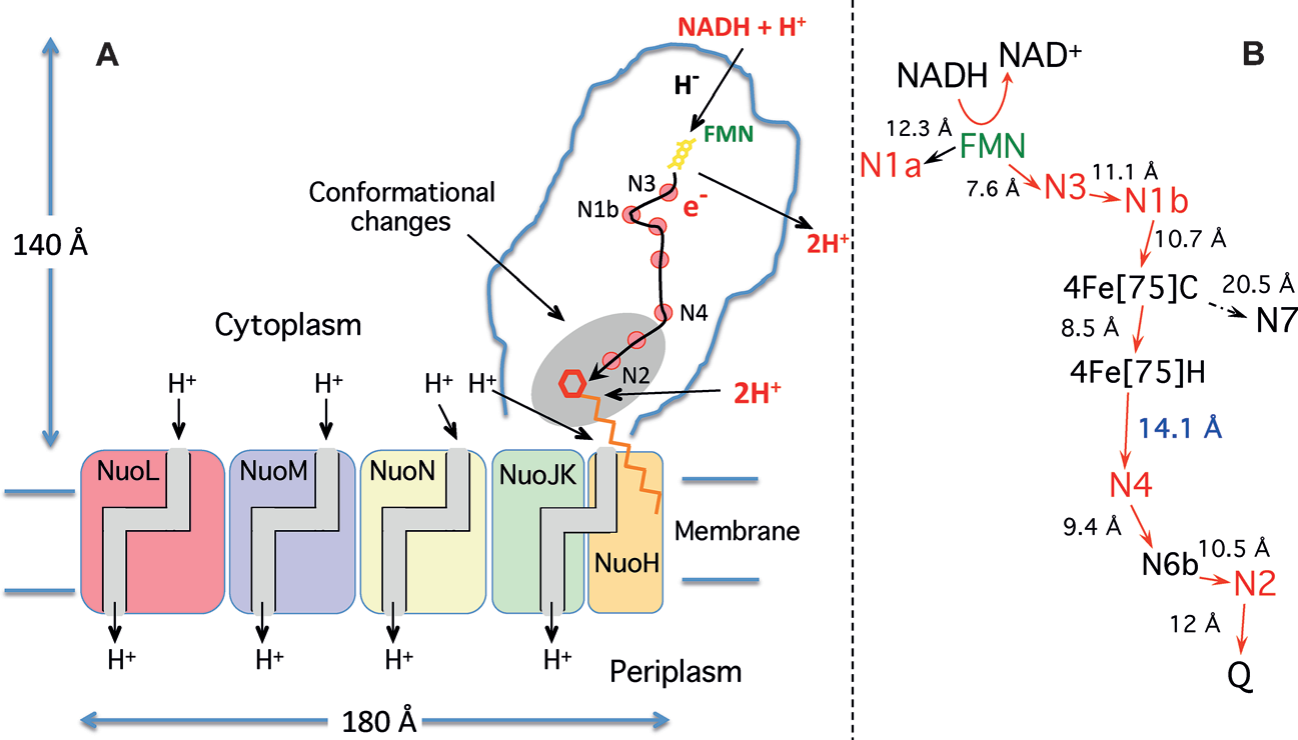
A) Scheme of complex I.[3b,4c,e] The red hexagon represents quinone. Gray area: region of redox-dependent conformational changes. B) Distances between the cofactors. FeS centers detected by EPR are shown in red. FMNH_2_ reduces N3, FMNH* reduces N1a. The distance from 4Fe[75]C to N7 (20.5 Å) is too long for NADH oxidation at 150–200 s^−1^.[5]

Here, we present a full quantitative analysis of the reaction between NADH and a highly pure preparation of the *E. coli* complex I[10] in the presence and absence of the Q-site inhibitor piericidin, while monitoring the redox states of both FMN and the FeS centers. Owing to differences in the freeze-quench methodology[11] both our experimental results and interpretation differ significantly from those in the previous publication.[9] The role of N2 in synchronizing electron tunneling and proton pumping rates is highlighted.

First, the number of NADH molecules oxidized by complex I (Figure S1) and the equilibrium electronic distribution within the complex were determined in the presence of piericidin to avoid reduction of endogenous Q (Figure S2). NADH is rapidly oxidized with a stoichiometry of 3.02±0.1 NADH per complex I. EPR spectroscopy shows an approximately equal distribution of four electrons in N1a (0.95±0.05), N1b (1.0±0.05), N2 (0.98±0.1), and N4 (0.90±0.1). N3 is reduced to 0.15±0.1 at most. Thus, all NADH-reducible FeS centers are EPR visible, and the other FeS centers (Figure 1) remain oxidized. Partial reduction of complex I by NADH is consistent with Mössbauer studies.[12] We further conclude that reduction of complex I is completed after three consecutive oxidations of NADH.

UV/Vis spectra of complex I reduced by NADH in the presence (Figure 2) and absence of piericidin (Figure S3) identify the FMN absorbance at 448 nm. In experiments with or without piericidin and using 100 mm and 2 mm NADH, respectively, the 448 nm peak was bleached within the first 97 μs of the reaction, indicating =85 % reduction of FMN, which remained fully reduced during the reaction. Note that reduction of the FeS centers occurs after a lag of ca. 100 μs following FMN reduction, indicated by their marginal reduction after 198 μs (Figure 2, S4). In the absence of piericidin, reduction of the FeS centers begins after 300–400 μs, which includes prior electron transfer to Q (Figure S3).

**Figure 2:**
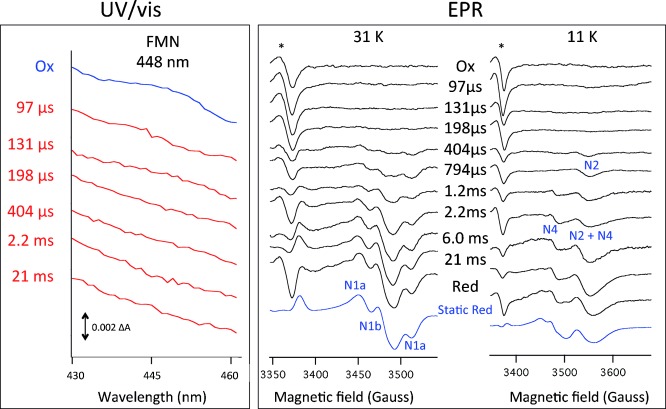
Low-temperature UV/Vis spectra highlighting the FMN spectral region (left) and EPR spectra (right) of complex I in the presence of piericidin freeze-quenched after different reaction times with 100 mm NADH. The EPR spectra show the *g_x_,g_y_* spectral range of the FeS centers. The asterisks indicate the *g*=2 radical region and the (variable) contribution due to the freeze-quench procedure. Static red: reduced by NADH and manually frozen. At 31 K centers N1a and N1b are seen, at 11 K centers N2 and N4 are also detectable.

The freeze-quench procedure produces small and variable amounts of radical(s) (*t*=0 traces (Ox), Figure 2 and Figure S3).[11a] However, the maximal amount of FMN radicals and Q radical was calculated as <0.04/enzyme, consistent with their low stability constants (*K*_stab_).[13] The observed multiphasic time course of FeS reduction (Figure 3 and Figure S5) is due to the three successive NADH turnovers and the particular thermodynamic values (*E*_m_) of the electron carriers. The experimental data (Figure 3 and Figure S5) were simulated in terms of the full reaction scheme (Figure 4) using the kinetic and thermodynamic parameters listed in Table 1. The *E*_m_ values of the FeS centers were calculated using *E*_m_ (FMN) and its *K*_stab_ as input parameters (Supporting Information). The simulation further includes the half-lives of FMN reduction, the lag period (100 μs), and, unexpectedly, two half-lives (200 μs and 1200 μs, Table 1) rather than one for the slowest electron-tunneling step detailed further below.

**Figure 3:**
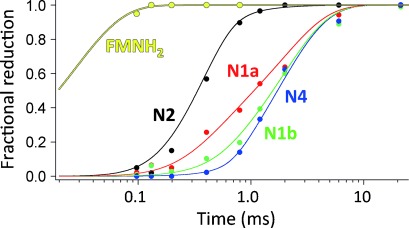
Kinetic profiles of FMN and the FeS centers in the presence of piericidin. Solid lines represent simulations on the basis of the kinetic scheme described in the text using the parameters in Table 1. FMN (yellow), N2 (black), N1a (red), N1b (green), and N4 (blue).

**Figure 4:**
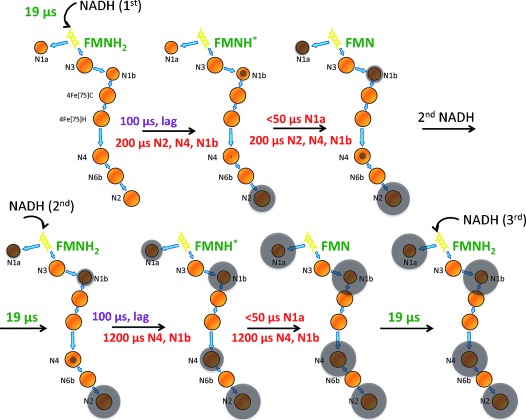
Reaction scheme for three sequential NADH turnovers by complex I in the presence of piericidin. The gray shaded circles indicate the degree of reduction of the particular FeS center. Note the sixfold difference in rates (*t*_1/2_=200 and 1200 μs) dependent on whether N2 is oxidized or reduced. FMN reduction occurs with *t*_1/2_=19 μs at [NADH]=100 mm. Dissociation of NAD^+^ occurs in the 100 μs lag period.

**Table 1:** Complex I kinetic and equilibrium constants.[a]

Center	*E*_m_ [mV] equil.[b]	*E*_m_ [mV] sim.[c]	*t*_1/2_ obs. [μs] N2 ox.	*t*_1/2_ obs. [μs] N2 red.
N2	−160	−159	200±20	
N1a	−330	−317	200±30	1200±200
N1b	−230	−223	200±20	1200±100
N4	−270	−257	200±20	1200±100
FMN→FMNH_2_	−259	−259	19±5[d]	19±5
lag period			100±20	100±20

	Half-lives of elementary reaction steps [μs]			
NADH→FMN (H^−^ transfer)	19±5[e]			
N4Fe[75]H→N4 (N2_ox_) N4Fe[75]H→N4 (N2_red_)	200 (±20) 1200 (±100)			
FMNH^*^→N1a	<50			
NAD^+^ dissociation	100±20			
protonation/deprotonation of FMNH_2_/FMNH^−^/FMNH^*^/N2	≪50			

[a] Data apply to pH 6, 10 °C.

[b] From equilibrium potentiometric titrations.[14]

[c] Calculated from simulation of the kinetic traces (Figure 3 and S5) using *E*_m,pH6_ (FMN/FMNH_2_)=−259 mV (Experimental Section) and *K*_stab_=4.5×10^−2^.[13b]

[d] At [NADH]=100 mm; *t*_1/2_=30 μs at 2 mm NADH.

[e] With *k*_on_ (NADH)=3.1±0.6×10^7^ m^−1^ s^−1^. Acceptable fits were obtained with the variation in rates indicated (±) and/or with the *E*_m_ values ±10 mV. All other elementary electron transfer steps are in the (sub)microsecond range.

The reduction of FMN to FMNH_2_ by the first NADH is followed by a 100 μs lag. The subsequent oxidation of FMNH_2_ to FMNH* leads to partial reduction of N1b, N4, and N2 according to their *E*_m_ values with *t*_1/2_=200 μs (Figures 2–4). The rapid initial reduction of N2 is consistent with its high *E*_m_ (Table 1), but that of N1a (Figures 2 and 3) is surprising because it has the lowest *E*_m_ of all FeS centers (Table 1).

The rapid reduction of N1a is explained by a rapid electronic equilibrium with the low-potential FMN/FMNH* redox couple (Table 1), whilst the chain of FeS centers between FMN and Q equilibrates with the FMNH*/FMNH_2_ couple. Since the initial reduction of N1a is as fast as that of N2, N1b, and N4 (Table 1), we conclude that the electronic equilibrium between N1a and FMNH* is very rapid (Figure 4), in agreement with the absence of a transient FMNH* radical (Figure 2 and Figure S3). Within the experimental uncertainties and a freeze-quench time of 50 μs,[11] the N1a–FMNH* equilibrium occurs with *t*_1/2_<50 μs (Table 1). The *E*_m_ values calculated from the electronic distribution of the FeS centers during the reaction closely match those determined by equilibrium potentiometric titrations[14] (Table 1) taking into account the differential equilibration of N1a with the FMN/FMNH* redox couple and the other FeS centers with the FMNH*/FMNH_2_ couple.

We expect that oxidation of the second NADH would show kinetics similar to those of the first, leading to an equal distribution of four electrons over N2, N1a, N1b, and N4. This leaves FMN formally oxidized (Figure 4). Rapid reduction of FMN by the third NADH marks the end of the reaction. This sequence of events was borne out experimentally, but, surprisingly, reduction of the remainder of the FeS centers by the second NADH was found to be sixfold slower (*t*_1/2_=1200 μs; Table 1, Figure 3) than by the first NADH.

The time course of FeS reduction in the absence of piericidin (Figures S3–S5) was simulated with the same set of kinetic and thermodynamic parameters (Table 1). Here, reduction of the FeS centers is in total delayed by 300–400 μs due to the lag period (*t*_1/2_=100 μs) and, more importantly, due to an initial reduction of Q (0.41 Q/complex I). The high *E*_m,pH6_ (Q)=150 mV prevents initial net reduction of the FeS centers (Table 1, Figure S5). Adequate simulation of the kinetic traces requires that both electrons that reduce Q travel with *t*_1/2_=200 μs (Figures S6 and S7). A simulation with *t*_1/2_=1200 μs for the second electron to Q, that is, when N2 is oxidized, produces a delay in FeS reduction that is inconsistent with the data (Figure S6).

We propose that *t*_1/2_=200 μs represents the elementary electron tunneling half-life across the 14.1 Å gap from 4Fe[75]H to N4. This half-life is within the range of the calculated values.[6–8] The *t*_1/2_=200 μs is observed for the first NADH turnover, both in the absence and presence of piericidin, specifically when N2 is oxidized (Figure 4, S7). The half-life for electron tunneling from 4Fe[75]H to N4 is increased to *t*_1/2_=1200 μs when N2 is reduced (Figure S7). Thus, the redox state of N2 determines the half-life of 4Fe[75]H to N4 electron tunneling and consequently the branching between the FMNH_2_→N2 and FMNH*→N1a pathways. When N2 is oxidized, both electrons travel from FMNH_2_ and FMNH* via N3 to N2. When N2 is reduced, FMNH_2_ reduces N1b and N4, whilst FMNH* reduces N1a in a ≈2/1 ratio between the two branches. The production of superoxide by complex I, which may lead to neurodegenerative diseases,[15] depends on the redox state of FMN,[16] which we propose is itself regulated by the redox state of N2.

Our data yield a NADH binding rate (*k*_on_) of 3.1±0.6×10^7^ m^−1^ s^−1^ in good agreement with *k*_cat_/*K*_M_ values (1.5–4.0×10^7^ m^−1^ s^−1^) from steady-state measurements[9,17] (Supporting Information) and an estimate for the half-life of hydride transfer of 20±5 μs that is consistent with the short distance for hydride transfer of 3.2 Å between the C^4N^ of the NADH nicotinamide ring and N^5^ of the FMN isoalloxazine ring, which are in stacking interaction.[4a]

The reaction with NADH comprises three sequential turnovers, yielding full reduction of FMN, N2, N1a, N1b, and N4 according to the following series of events: NADH reduces FMN to FMNH^−^ through hydride transfer followed by rapid protonation to FMNH_2_. Electron transfer from FMNH_2_ occurs in two single-electron-transfer steps with FMNH* as the intermediate and is preceded by non-rate-limiting (*t*_1/2_<50 μs, Table 1) deprotonation to the corresponding anions. Oxidation of FMNH_2_ occurs after a lag of 100 μs ascribed to dissociation of NAD^+^. FMN is a branching point for electron transfer either towards Q or N1a dependent on the redox state of N2 (Figure S7). Electron transfer from FMNH_2_ (and/or FMNH*) to Q occurs with *t*_1/2_=200 μs when N2 is oxidized. Reduction of N2 is accompanied by (fast) protonation since its *E*_m_ depends on pH.[14] When N2 is reduced, electron transfer is decelerated to *t*_1/2_=1200 μs. This sixfold slower reduction cannot be explained by a slow millisecond dissociation[9] of NAD^+^ before the second NADH binds, because FMN remains reduced during the reaction (Figure 2 and Figure S3). This further indicates that dissociation of NAD^+^ is faster than the limiting electron transfer of 200 μs. We propose that dissociation of NAD^+^ occurs in the lag period before the onset of FeS reduction; its estimated *t*_1/2_=100 μs is consistent with the value of <140 μs calculated from steady-state rate measurements.[13a,^18^] The minimal kinetic scheme (Figure 4) assumes rapid electronic equilibrium between all redox centers (Table 1). This assumption is justified, since most elementary electron transfer steps occur within the two electron-tunneling half-lives determined in this work (*t*_1/2_=200 and 1200 μs) and even within the experimental freeze-quenching time of ca. 50 μs[11] The condition of rapid equilibrium prevents detection of very short-lived intermediates states, such as transiently reduced FeS centers (Figure 4). Furthermore, FMN or Q radicals do not accumulate owing to their low stability constants and the rapid equilibration with their respective direct redox partners, N1a (<50 μs) and N2 (≈0.4 μs[7]). As a result of the rapid equilibrations, all FeS centers follow the same time course of reduction given by the two longest electron tunneling half-lives.

The sixfold increase in electron tunneling time is ascribed to a sixfold slower electron transfer from 4Fe[75]H to N4 (Figure 1). For any other elementary reaction the change in rate would have to be =1000 fold and give rise to a different electronic distribution over the FeS centers than that observed and simulated. According to the Marcus equation[19] a sixfold change in *k*_ET_ can be obtained by 1) a sixfold change of *V*_0_^2^, the square of the maximal electronic coupling between 4Fe[75]H and N4; 2) a change in the distance between the FeS centers of ca. 1.3 Å; 3) a change by ca. 0.2 eV in the reorganization energy *λ*; or 4) a change in Δ*G*^0^ of ca. 0.1 eV. A change in distance is unlikely in view of the similar structures of the oxidized and reduced enzyme,[4a] also making a change in *λ* less likely. A change in Δ*G*^0^ can be obtained by increasing the *E*_m_ of N4 by roughly 0.1 eV. However, this would bring the *E*_m_ of N4 close to that of N2 (Table 1) and predicts similar initial reduction kinetics for N4 and N2, in contrast to observation (Figures 2 and 3). Conversely, the *E*_m_ of 4Fe[75]H might be lowered but this effect was calculated as negligible.[20]

The electron transfers N3 to N1b, 4Fe[75]H to N4, and N6b to N2 occur across subunit boundaries. The calculated electron transfer rates between these centers are strongly dependent on the presence of water at the subunit boundary.[7] Water is an essential mediator[21] increasing the rate of electron transfer by increasing *V*_0_^2^ by factors of roughly 2400, 700, and 1000 for these three respective electron transfers.[7] Thus, the 4Fe[75]H to N4 electron transfer can be slowed down by small changes in the structure of interfacial water, caused, for example, by a slightly different relative arrangement of two subunits. Crystallographic analyses indicate that the coupling of electron transfer to proton pumping is due to conformational changes driven by the redox chemistry of N2 and Q that are transmitted to four proton channels in the membrane arm.[3b,4a,b,f] We propose that the conformational changes triggered by reduction of N2 decrease the electronic coupling, thus tuning the 4Fe[75]H to N4 electron tunneling to the millisecond time domain in order to synchronize electron transfer with proton pumping. Indeed, electron transfer and proton pumping are calculated to proceed at similar rates during in vivo steady-state turnover in *E. coli* where N2 is reduced (Supporting Information). Synchronization of electron transfer with proton-pumping reactions is an important means to minimize the dissipation of redox free energy and to optimize the mechanistic coupling and, hence, the efficiency of energy transduction. Thus, the chain of iron–sulfur centers is not just a simple electron-conducting wire; it also modulates the electron-tunneling rate during the reaction.

In order to control electron transfer rates from nanoseconds to milliseconds a chain of three or four FeS centers might suffice,[22] as, for example, found in succinate dehydrogenase,[22a] fumarate reductase,[22b] formate dehydrogenase,[22c] nitrate reductase,[22d] hydrogenase,[22e] and nitrogenase.[22] In these enzymes electron transfer is coupled to protonation, which must be properly matched to prevent formation of energetically unfavorable intermediates slowing down catalysis or avoid production of highly reactive intermediates. Proper timing is achieved by redox tuning, lowering the *E*_m_ of the central FeS center slowing down electron transfer to milliseconds.[6] Redox tuning was proposed as a mechanism to prevent the formation of toxic singlet oxygen species[23] by the long photosynthetic electron transfer chains. Long redox chains provide the structural basis and Marcus theory the theoretical basis for nature to exploit simple biophysical principles to vary electron transfer rates over a wide range by tuning distances, driving forces, and electronic couplings to evolve efficient and specific biocatalysts and a highly efficient energy converter, respiratory complex I.
