# Supplementary material for: Electron Tunneling Rates in Respiratory Complex I Are Tuned for Efficient Energy Conversion
Source: Angew Chem Int Ed Engl. 2015 Jan 19;54(9):2844–8. doi: 10.1002/anie.201410967 (PMC4506566; doi:10.1002/anie.201410967)
Supplement: Supplementary file 1 [file anie0054-2844-sd1.pdf]

Angewandte Chemie
Eine Zeitschrift der Gesellschaft Deutscher Chemiker

Supporting Information

© Wiley-VCH 2015

69451 Weinheim, Germany

Electron Tunneling Rates in Respiratory Complex I Are Tuned for Efficient Energy Conversion**

Simon de Vries, Katerina Dörner, Marc J. F. Strampraad, and Thorsten Friedrich**

anie_201410967_sm_miscellaneous_information.pdf

SUPPORTING INFORMATION

Contents

Supporting Materials and Methods

General

Midpoint potential of FMN.

Freeze-quench studies.

Data treatment.

Kinetic simulations.

Hydride transfer rate

Synchronization of electron transfer and proton pumping.

Supporting Figures (S1 – S7)

Supporting References

Supporting Materials and Methods

General Complex I purification and activity measurements (k_{cat}= 150 s^{-1}) were as described previously [1]. FMN (0.98 ± 0.04 per complex I) and Q (0.41 ± 0.04 per complex I) were determined as described in [2], respectively. The Marcus expression for the electron-tunnelling rate is given by [3]: $k_{ET}\ (s^{-1}) = \frac{2\pi}{\hbar}\frac{V_R^2}{\sqrt{4\pi\lambda k_B T}}.\exp[\frac{-(\lambda+\Delta G^0)^2}{4\lambda k_B T}]$. Herein λ is the reorganization energy, ΔG^0 the driving force and V_R^2 the electronic coupling containing the distance dependence as: $V_R^2 = V_0^2 \exp(-\beta R)$, with β the exponential decay coefficient, R, the donor-acceptor distance and V_0^2, the maximum electronic coupling [3b].

Midpoint potential of FMN. The midpoint potential of FMN at pH 6 was determined kinetically at E_m= -259.0 ± 2.0 mV. To this end, the activity was determined at fixed [NADH]= 160 μM and variable [NAD^+] up to 2 mM. The observed rates at the different NAD^+/NADH ratios were fitted to the Nernst equation.

Freeze-quench studies. Complex I (27.3 μM) was incubated on ice for 2 minutes with a buffer of 50 mM MES/NaOH, 50 mM NaCl, 0.1% lauryl maltoside, 10 mg/ml *E. coli* polar lipid extract (Avanti), pH 6, in a 1:1 ratio. This mixture was rapidly mixed 1:1 with the same buffer containing NADH yielding final concentrations of 2, 5 or 100 mM NADH and 6.8 μM Complex I, rapidly frozen and analyzed by EPR spectroscopy and low-temperature UV-vis spectroscopy (110 K) as described previously [4]. EPR experiments were performed on a Bruker EC106 EPR spectrometer. EPR conditions: Microwave frequency, 9.45 GHz; modulation amplitude, 1.0 mT; microwave power, 10 mW at 11 K and 2 mW at 31 K.

Data treatment. Kinetic experiments were performed in duplicate or triplicate producing a 0.07 standard error of the mean in the relative concentrations of the FeS centres displayed by the error bars in Fig. S5. Owing to the loose packing (~20%) of the freeze-quenched samples [4], the effective complex I concentration in EPR tubes is ~ 1.3 μM. This led to broad baseline features due to trapped oxygen in the g_z spectral region of the FeS centers, and thus only the g_x, g_y region was analyzed. EPR spectra were averaged three times. The degree of reduction of the FeS centers (N1a/N1b and N2/N4) was determined using the spectral simulations shown in Fig. S2 by application of a home-written two-component analysis procedure written

in the software program IGOR Pro (6.06), Wavemetrics, Inc. (OR, USA) using its incorporated root-mean-square minimization routine.

Kinetic simulations. Kinetic simulations were performed with a home-written program that numerically calculates up to 12 consecutive reversible first order reactions and that runs under IGOR Pro (6.06). This calculation yields the overall time course of reduction of enzyme intermediate states (defined below), i.e. the total number of electrons per complex I in a particular state. The electronic distribution over the FeS (N1a, N1b, N2, N4) per state was subsequently calculated in a separate program using as input the determined $E_{m,pH6}$ (FMN/$FMNH_2$) = -259 mV and K_{stab} = 4.5 10^{-2} from [5] in order to obtain the midpoint potentials of the FeS centers, and their individual time courses. Specifically, N1a was taken to equilibrate with the FMN/FMNH* redox couple, the other FeS centers with the FMNH*/$FMNH_2$ redox couple. The simulations were manually iterated until the simulated traces were within experimental error. Given the experimental error in the redox state of the FeS centers (SEM = 0.07) parameter variation analyses yield a variation in E_m of ± 10 mV quoted in Table 1 for acceptable fits to the data.

The reaction with NADH comprises three sequential turnovers depicted in Fig. 4 leading to the full reduction of FMN, N2, N1a, N1b, and N4. The simulation includes the half-lives of FMN reduction (19 μs (Fig. 3) or 30 μs (Figs. S4 and S5)), the lag period (100 μs) representing dissociation of NAD^+, and two half-lives (200 μs to 1200 μs) representing electron tunneling from 4Fe[75]H → N4 dependent on whether N2 is oxidized or reduced, respectively.

The time courses of FeS reduction with or without piericidin (Fig. 3 and Fig. S5) use the same half-lives. Given the Q content (0.41 ± 0.04 per complex I) simulation of the data in the absence of piercidin were performed as follows. The Q-content implies that 59% of complex 1 is not associated with Q, and reacts in the same manner as in the presence of piericidin, i.e. no electron transfer to Q (Fig. 5). The 41% of complex 1 with associated Q actually makes four NADH turnovers. In the first of these turnovers, reduction of the FeS centers is ~ zero given the $E_{m,pH6}$ (Q/QH_2) = 150 mV. Further, both electrons from $FMNH_2$ are transferred to Q with $t_{1/2}$ = 200 μs. A calculation in which the first electron has $t_{1/2}$ = 200 μs and the second $t_{1/2}$ = 1200 μs, does not fit the data (Fig. S6). The absence of initial reduction of the FeS leads to an apparent total delay (i.e. including 100 μs of NAD^+ dissociation) of 300 - 400 μs for N2 reduction (Fig. S5, compare black and black dotted traces).

At [NADH] = 100 mM (Figs. 2 and 3) and 6.8 μM complex I, the final redox potential ($E_{m,pH6}$ (NAD^+/NADH) = -290 mV; 10°C) is -497 mV, making the overall reaction essentially irreversible and leading to full reduction of the FeS centers. Since [NADH]>> complex I, FMN reduction is pseudo-first-order, reduction of the FeS first-order. The three consecutive turnovers can be written in reaction equation with the enzyme intermediate states E, E', E'', E''' and E'''' as:

E + 1stNADH = E(NADH) = E'(NAD^+) = E' +NAD^+ = E''+ 2ndNADH = E''(NADH) = E''' (NAD^+) = E''' + NAD^+ = E'''' + 3rdNADH = E''''(NADH) = E'''''(NAD^+) = E'''' + NAD^+

Herein, E, E', E'', E''', E'''' and E''''' are the oxidized complex 1 and reduced with 2, 2, 4, 4 and 6 electrons, respectively. The scheme indicates 1) Binding of the 1st NADH; 2) FMN reduction by hydride transfer; 3) Dissociation of NAD^+; 4) Electron transfer from flavin to the FeS centers (E' to E''). These steps are repeated for binding of the 2nd NADH, and the reaction stops after binding the 3rd NADH, reduction of FMN and dissociation of NAD^+. The time courses of the intermediate states are calculated first and subsequently the electronic distribution as described above.

Hydride transfer rate. The half-lives (30 μs and 19 μs) determined for FMN reduction at 2 mM and 100 mM NADH, respectively, yield a NADH binding rate (k_{on}) of 3.1 ± 0.6 10^7 $M^{-1}s^{-1}$ and a half-life for hydride transfer from NADH to FMN of 20 ± 5 μs. The value of k_{on} is in good agreement with calculations of k_{cat}/K_M (1.5 – 4.0 10^7 $M^{-1}s^{-1}$) based on the value of K_M for NADH, 5 - 10 μM [6] and on k_{cat} = 150 - 200 s^{-1} determined by steady-state measurements [6-7].

Synchronization of electron transfer and proton pumping. The turnover rate or k_{cat} = 150 - 200 s^{-1} calculates to τ = 5 – 6.7 ms per NADH. The slowest electron transfer with $t_{1/2}$ = 1200 μs yields τ = 1.73 ms. This implies τ = 3.5 ms per NADH for steady-state turnover when N2 is reduced as occurs in *E. coli in vivo* [8]. With respect to the overall rate, the rate of electron transfer is approximately half the total rate, implying that electron transfer and proton transfer/proton pumping rates are very similar. This calculation assumes that Q binding and QH_2 release are fast compared to proton transfer/proton pumping.

Supporting Figures

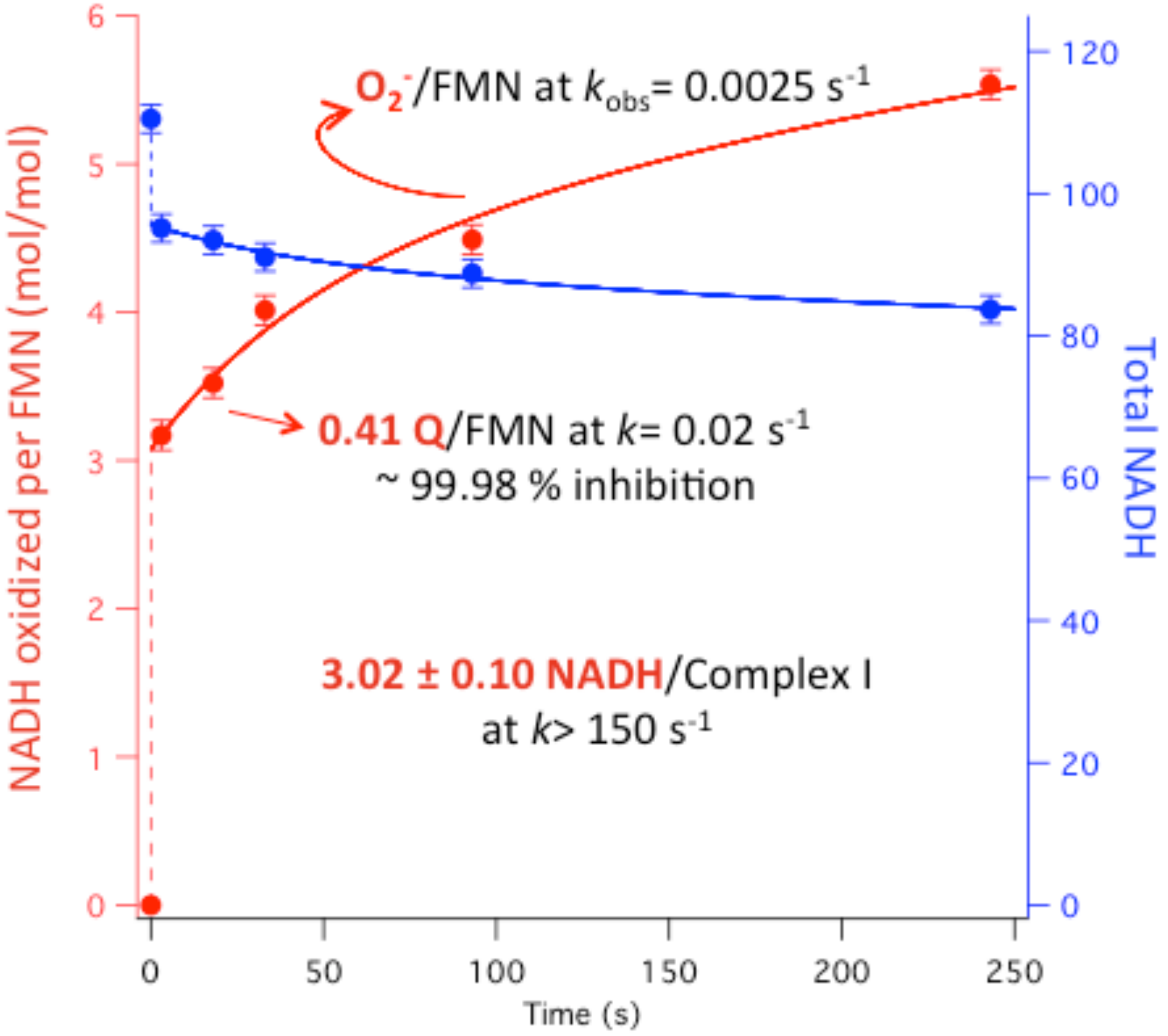

Figure S1. **Steady-state near anaerobic (3 µM O_2) oxidation of NADH in the presence of piericidin.** In the rapid initial phase, which is not time resolved, a stoichiometry of 3.02 ± 0.1 NADH per complex I is determined. Reduction of Q at the longer time scale accounts for the first order reduction of the 0.41 Q/complex I at a rate of 0.02 NADH/s implying 99.98% inhibition by piercidin. Complex I is known to generate superoxide at a rate of ~ 0.2/s in normoxic conditions [7a] (~ 250 µM O_2), which at 3 µM O_2 of the experiment results in an apparent rate of 0.0025/s used in the simulation of the kinetic trace.

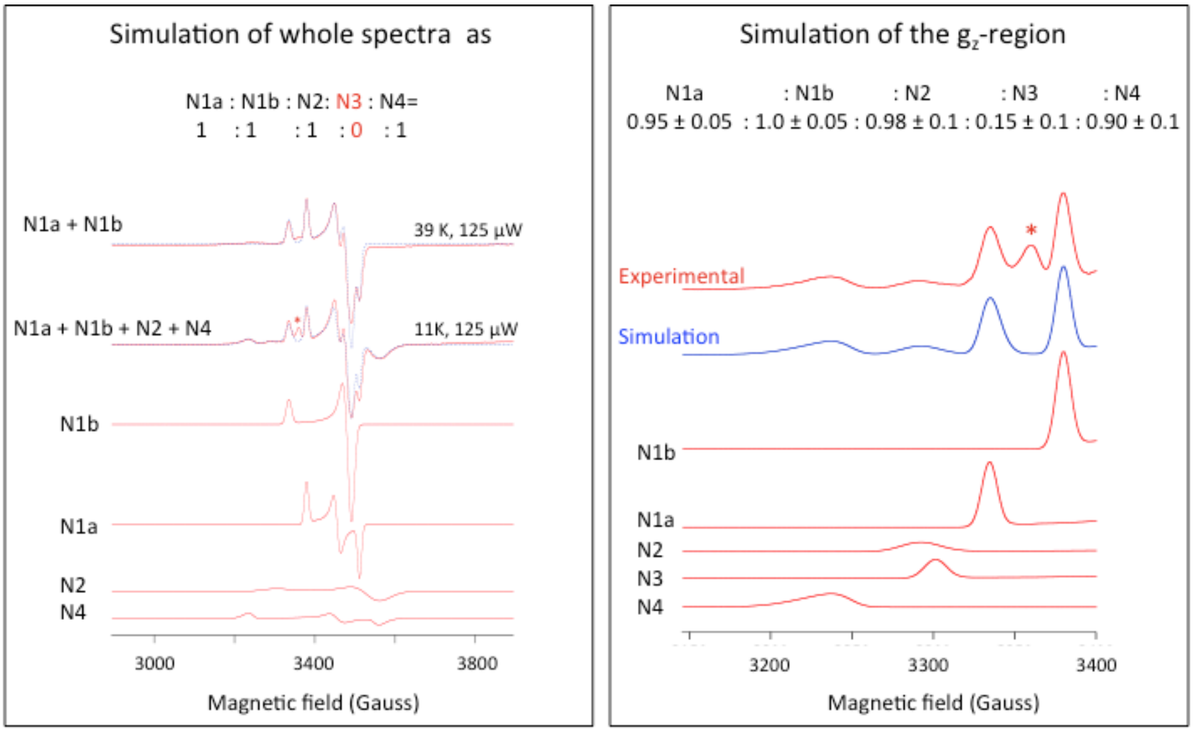

Figure S2. EPR spectra of complex I reduced with NADH (2 mM) and calculation of the distribution of four electrons over the FeS centers using spectral simulation employing two methods. Whole spectrum simulation with equal distribution over the FeS centers N1a, N1b, N2 and N4 (left panel); the more accurate analysis using the g_z resonances (right panel) enabling an estimate for N3, suggests a very similar near equal stoichiometry. For kinetic analyses (cf. Table 1) an equal ratio was used: N1a: N1b: N2: N4 = 1: 1: 1: 1. EPR simulation parameters: N1a: $g_{z,y,x}$= 1.9938, 1.9499, 1.91903. Line width (W): $W_{z,y,x}$= 10.5, 11.1, 14.0 Gauss. N1b: $g_{z,y,x}$= 2.0200, 1.9370, 1.92969. Line width (W): $W_{z,y,x}$= 11.0, 12.0, 14.0 Gauss. N2: $g_{z,y,x}$= 2.0501, 1.909, 1.904. Line width (W): $W_{z,y,x}$= 14.0, 24.0, 24.0 Gauss. N4: $g_{z,y,x}$= 2.0850, 1.950, 1.890. Line width (W): $W_{z,y,x}$= 15.0, 15.0, 15.0 Gauss. Strain parameters for line width: Wxy= 22.5 Gauss; Wxz= 0; Wyz= 15 Gauss. N3 (right panel only, and displayed at the same concentration as N1b): g_z= 2.0455. Line width (W): W_z= 15.8 Gauss. The asterisk indicates a radical species of unknown origin. Spectra were recorded at non-saturating microwave powers (125 µW) for both 31 K and 11 K.

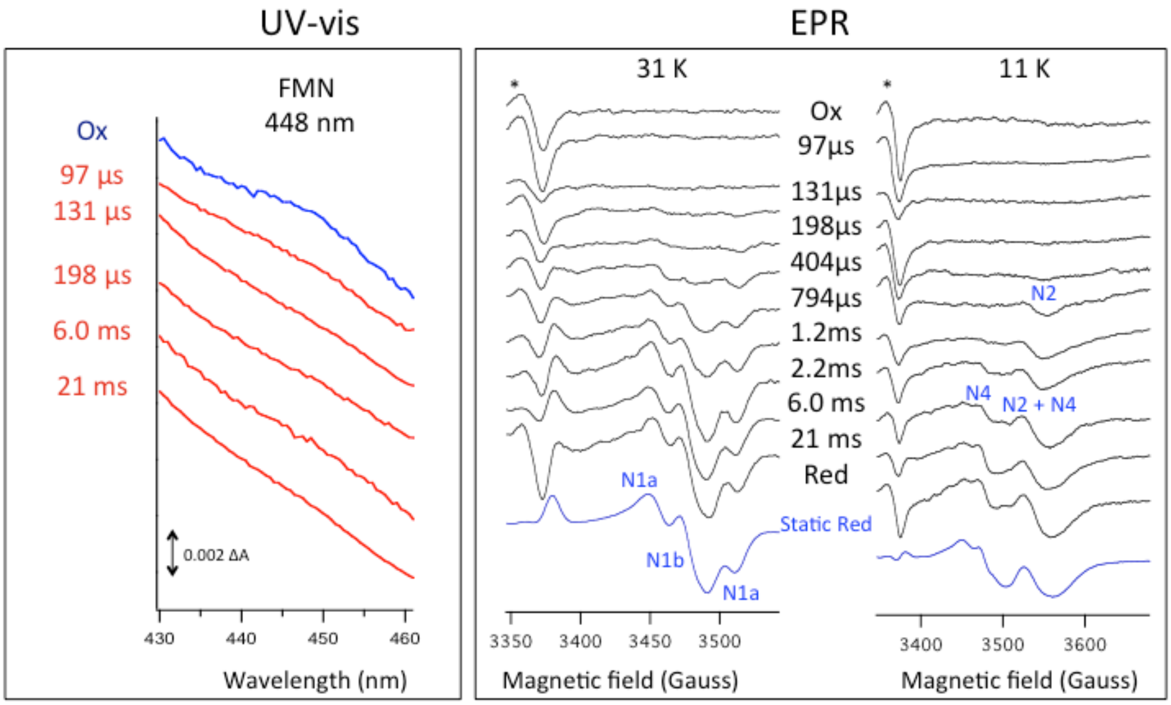

Figure S3. Low temperature Uv-vis spectra (left panel) and EPR spectra (at 31K and 11K, right panel) of complex I after different reaction times with 2 mM NADH in the absence of piericidin. Ox (t = 0) and Red, samples prepared in the absence of or pre-reduced by NADH, respectively, before freeze-quenching; Static red, reduced by NADH and manually frozen. UV-vis spectra of oxidized complex I show the peak from FMN at 448 nm. The EPR spectra show the g_x, g_y spectral range and the specific resonances of the FeS centers. The asterisk indicates the g = 2 radical region and the (variable) contribution due to the freeze-quench procedure (see e.g. Ox). At 31 K centers N1a and N1b are seen, at 11K centers N2, N4 and partially N3.

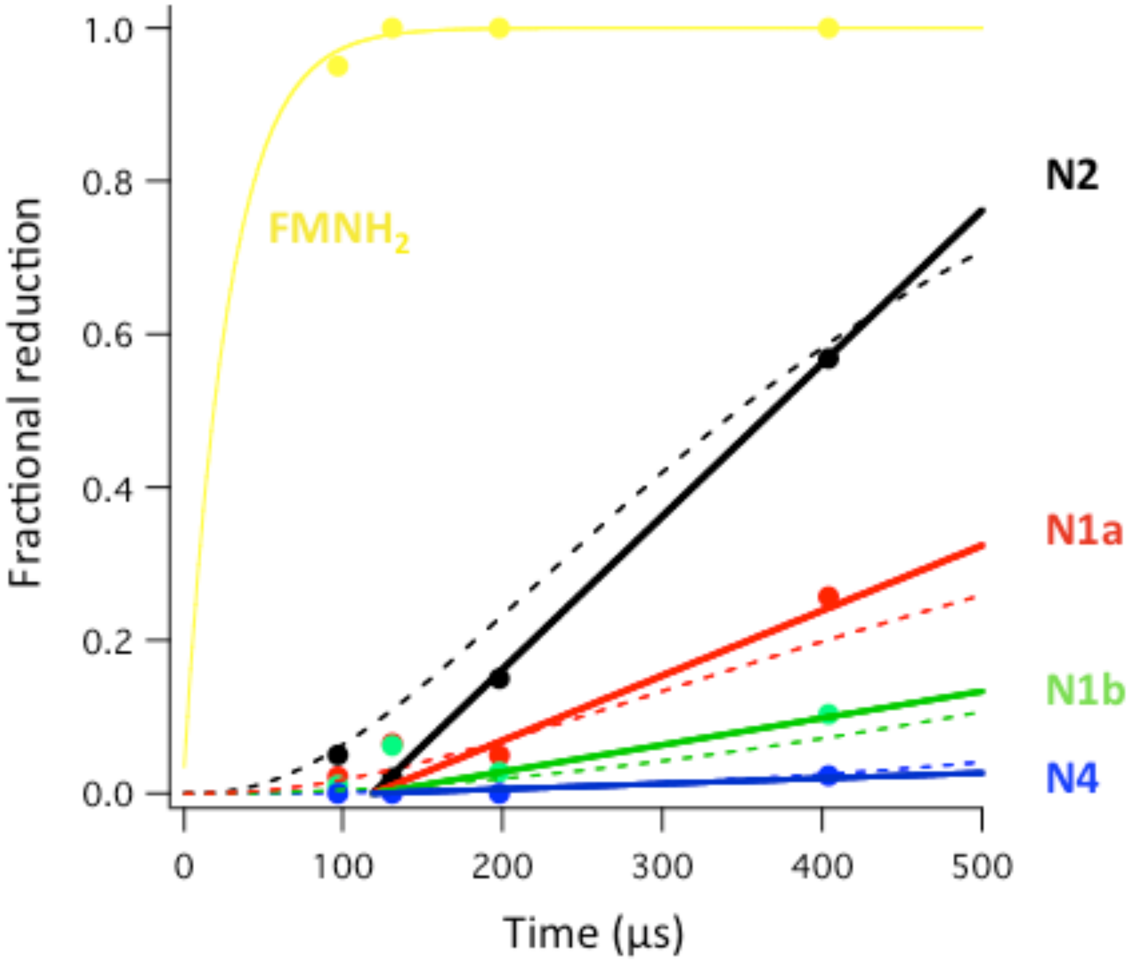

Figure S4. Graphical analysis to estimate the lag period observed for the reduction of the FeS centers of complex 1 in the presence of piericidin. Data obtained at 100 mM NADH and simulations as in Fig. 3. Dotted lines are the simulated traces (with a lag of 100 µs as in Fig. 3). Straight lines through the data points obtained after 198 and 404 µs converge at t = 119 µs. Given that FMN reduction takes 19 µs, the lag for reduction of the FeS centers is ~100 µs, a value that yields satisfactory fits to the data (dotted lines), and Fig. 3.

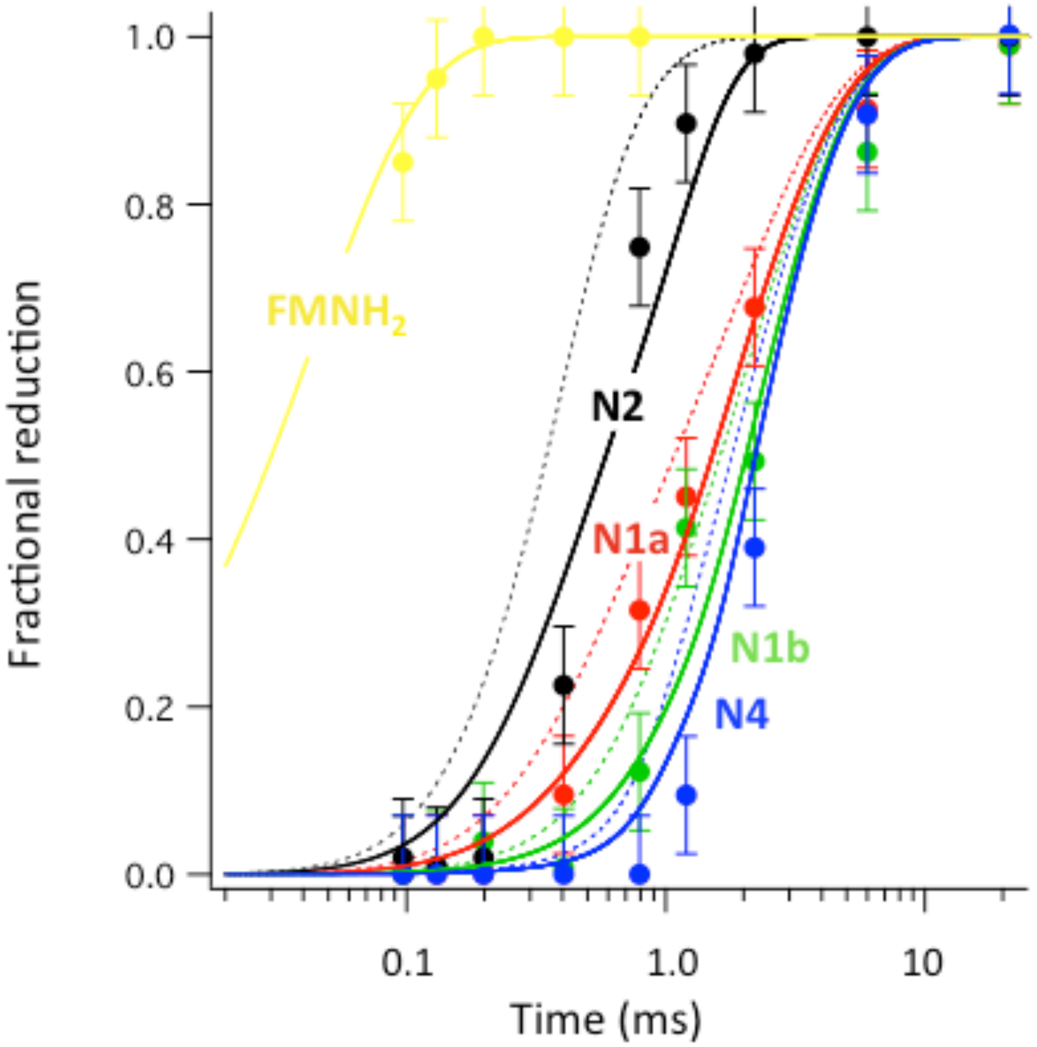

Figure S5. Simulation of the time course of reduction of FMN and the FeS centers. Data and simulations for the reaction in the absence of piericidin; dotted lines, simulation of the kinetics in the presence of piericidin (as in Fig. 3). Simulations based on half-lives listed in Table 1, and for data in the absence of piericidin taking into account the amount of 0.41Q/complex 1, and both electrons traveling with $t_{1/2}$ = 200 μs in the first turnover (see Figs. 4 and 5). N2 (●), N1a (●), N1b (●), and N4 (●). The SEM (0.070) is indicated in the figure and applies also to Fig. 3, and Figs. S4 and S6.

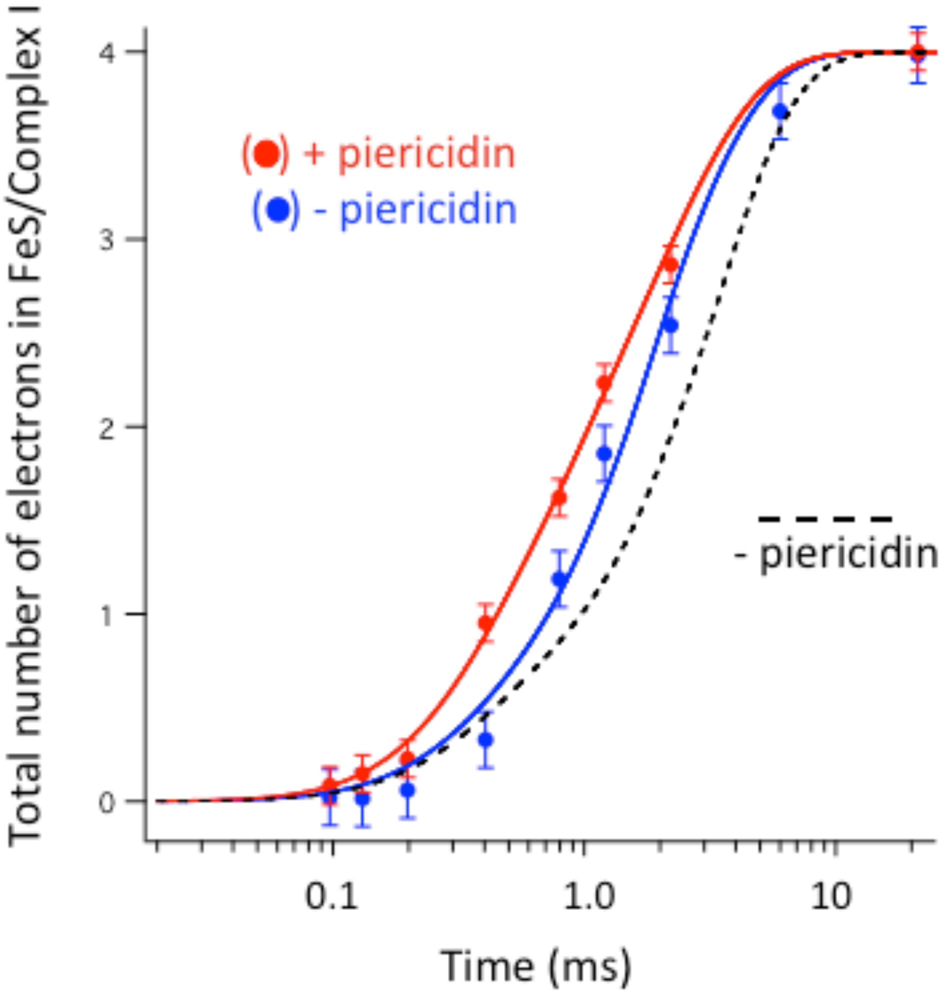

Figure S6. Total number of electrons in the FeS centers and simulations (solid lines) for the reduction of complex I in the presence (red) or absence (blue) of piericidin. Data were used from Figs. 2 and S3 and their respective simulations (Fig. 3 and Fig. S5). The longer lag in the absence of piericidin is due to initial reduction of Q whilst FeS centers remain oxidized. The dotted line represents a simulation to the data in the absence of piericidin, assuming that in the first NADH turnover the first electron travels with $t_{1/2} = 200$ µs and the second with $t_{1/2} = 1200$ µs (and not with $t_{1/2} = 200$ µs). The lack of correspondence to the data (blue circles) indicates that both electrons travel with $t_{1/2} = 200$ µs, as long as N2 remains oxidized (Figs. 4, 5 and S5).

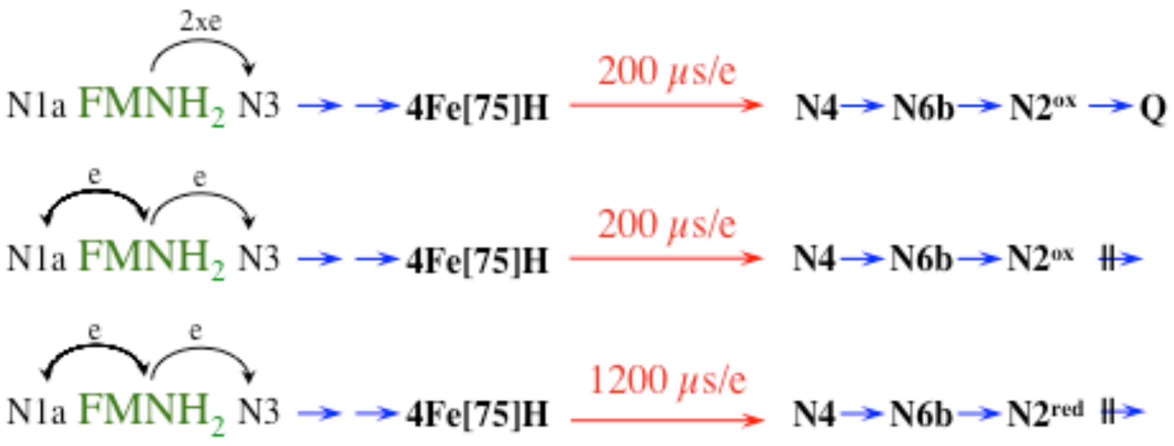

Figure S7. The redox state of N2 determines the electron tunneling half-live from 4Fe[75]H to N4 and the electronic distribution at FMN. In the absence of piericidin (upper drawing) electrons from $FMNH_2$ travel one-by-one to Q with $t_{1/2}$ = 200 μs because N2 remains oxidized. In the presence of piericidin (||), $FMNH_2$ to N2 electron transfer occurs with the same $t_{1/2}$ = 200 μs (middle drawing). With N2 reduced (lower drawing) electron tunneling occurs with $t_{1/2}$ = 1200 μs. The curved arrows at $FMNH_2$ indicate the potential branching of electron transfer pathways; straight arrows indicate direct pathways.

Supporting references

[1] a T. Pohl, M. Uhlmann, M. Kaufenstein, T. Friedrich, *Biochemistry* **2007**, *46*, 10694-10702; b S. Stolpe, T. Friedrich, *J Biol Chem* **2004**, *279*, 18377-18383.

[2] a M. D. Miramar, P. Costantini, L. Ravagnan, L. M. Saraiva, D. Haouzi, G. Brothers, J. M. Penninger, M. L. Peleato, G. Kroemer, S. A. Susin, *J Biol Chem* **2001**, *276*, 16391-16398; b A. Kroger, M. Klingenberg, *Biochemische Zeitschrift* **1966**, *344*, 317-336.

[3] aR. A. Marcus, N. Sutin, *Biochim Biophys Acta* **1985**, *811*, 265-322; bC. C. Moser, J. M. Keske, K. Warncke, R. S. Farid, P. L. Dutton, *Nature* **1992**, *355*, 796-802.

[4] a A. V. Cherepanov, S. De Vries, *Biochim Biophys Acta* **2004**, *1656*, 1-31; b F. G. Wiertz, O. M. Richter, B. Ludwig, S. de Vries, *J Biol Chem* **2007**, *282*, 31580-31591.

[5] a J. A. Birrell, G. Yakovlev, J. Hirst, *Biochemistry* **2009**, *48*, 12005-12013; b T. Ohnishi, S. T. Ohnishi, K. Shinzawa-Itoh, S. Yoshikawa, R. T. Weber, *Biochim Biophys Acta* **2012**, *1817*, 1803-1809; c V. D. Sled, N. I. Rudnitzky, Y. Hatefi, T. Ohnishi, *Biochemistry* **1994**, *33*, 10069-10075.

[6] M. L. Verkhovskaya, N. Belevich, L. Euro, M. Wikstrom, M. I. Verkhovsky, *Proceedings of the National Academy of Sciences of the United States of America* **2008**, *105*, 3763-3767.

[7] a L. Kussmaul, J. Hirst, *Proceedings of the National Academy of Sciences of the United States of America* **2006**, *103*, 7607-7612; b S. Steimle, C. Bajzath, K. Dorner, M. Schulte, V. Bothe, T. Friedrich, *Biochemistry* **2011**, *50*, 3386-3393.

[8] M. R. de Graef, S. Alexeeva, J. L. Snoep, M. J. Teixeira de Mattos, *Journal of bacteriology* **1999**, *181*, 2351-2357.
